# Atrioventricular Area Difference Aids Diastolic Filling in Patients with Repaired Tetralogy of Fallot

**DOI:** 10.1007/s00246-024-03508-7

**Published:** 2024-05-28

**Authors:** Martin Johansson, Erik Hedström, Katarina Steding-Ehrenborg, Misha Bhat, Petru Liuba, Håkan Arheden, Pia Sjöberg

**Affiliations:** 1 Clinical Physiology, Department of Clinical Sciences Lund, Lund University, Lund, Sweden; 2 Department of Clinical Physiology, Skåne University Hospital, 22185 Lund, Sweden; 3 Department of Pediatric Anesthesia and Intensive Care, Skåne University Hospital, Lund, Sweden; 4 Diagnostic Radiology, Department of Clinical Sciences Lund, Lund University, Lund, Sweden; 5 Department of Radiology, Skåne University Hospital, Lund, Sweden; 6 Pediatrics, Department of Clinical Sciences Lund, Lund University, Lund, Sweden; 7 Pediatric Cardiology, Children’s Heart Centre, Skåne University Hospital, Lund, Sweden

**Keywords:** Congenital heart defect (CHD), Tetralogy of Fallot, Diastolic function, Cardiac magnetic resonance (CMR), Hydraulic force, Physiology

## Abstract

A hydraulic force aids diastolic filling of the left ventricle (LV) and is proportional to the difference in short-axis area between the left ventricle and atrium; the atrioventricular area difference (AVAD). Patients with repaired Tetralogy of Fallot (rToF) and pulmonary regurgitation (PR) have reduced LV filling which could lead to a negative AVAD and a hydraulic force impeding diastolic filling. The aim was to assess AVAD and to determine whether the hydraulic force aids or impedes diastolic filling in patients with rToF and PR, compared to controls. Twelve children with rToF (11.5 [9–13] years), 12 pediatric controls (10.5 [9–13] years), 12 adults with rToF (21.5 [19–27] years) and 12 adult controls (24 [21–29] years) were retrospectively included. Cine short-axis images were acquired using cardiac magnetic resonance imaging. Atrioventricular area difference was calculated as the largest left ventricular short-axis area minus the largest left atrial short-axis area at beginning of diastole and end diastole and indexed to height (AVADi). Children and adults with rToF and PR had higher AVADi (0.3 cm^2^/m [− 1.3 to 0.8] and − 0.6 [− 1.5 to − 0.2]) at beginning of diastole compared to controls (− 2.7 cm^2^/m [− 4.9 to − 1.7], *p* = 0.015) and − 3.3 cm^2^/m [− 3.8 to − 2.8], *p* = 0.017). At end diastole AVADi did not differ between patients and controls. Children and adults with rToF and pulmonary regurgitation have an atrioventricular area difference that do not differ from controls and thus a net hydraulic force that contributes to left ventricular diastolic filling, despite a small underfilled left ventricle due to pulmonary regurgitation.

## Introduction

Tetralogy of Fallot is the most common, complex congenital heart defect requiring surgery at an early age. Although initial mortality is only 1.1–2.5% [1–3], and a majority of patients reach adulthood, subsequent morbidity and particularly pulmonary regurgitation is still common and several patients develop heart failure [4–8]. Left ventricular diastolic dysfunction is also common after corrective surgery [9–11] and is a predictor of poor outcome [5] as well as indicative of greater risk of major adverse events [12]. However, the importance of atrioventricular interaction in the setting of pulmonary regurgitation and diastolic dysfunction is not fully understood in patients with repaired Tetralogy of Fallot (rToF).

**Fig. 1:**
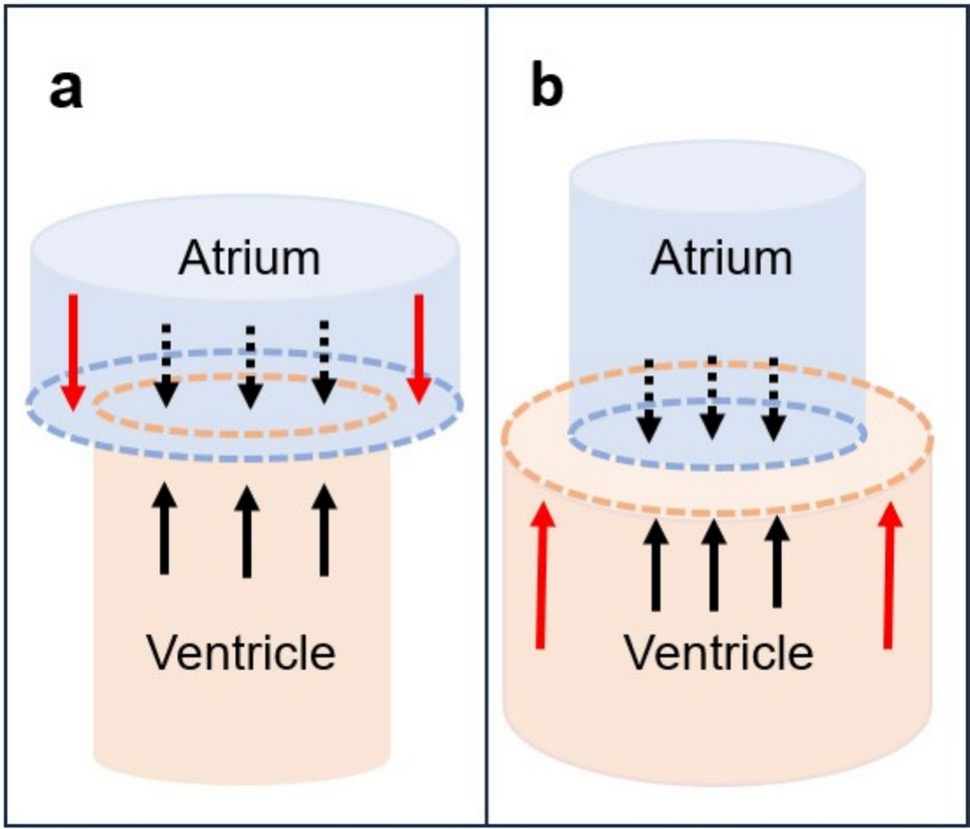
Schematic illustration of the left side of the heart describing the atrioventricular area difference. **a** shows the heart at the beginning of diastole with a larger atrium and smaller ventricle. **b** shows the heart at the end of diastole with a smaller atrium and larger ventricle and thus a hydraulic force contributing to diastolic filling. Arrows represent the force of the blood acting upon the atrioventricular plane. Solid black and dashed black arrows counterbalance each other while the red arrows represent the net force acting on the atrioventricular plane. The red arrows indicate the direction of the net hydraulic force. The magnitude of the hydraulic force is proportional to the atrioventricular area difference (AVAD) calculated as; *ventricular short-axis area—atrial short-axis area*. An AVAD less than zero will yield a net hydraulic force acting toward the apex of the heart while an AVAD larger than zero corresponds to a hydraulic force pushing the atrioventricular plane away from the apex of the heart

The geometrical relationship between the left atrium and ventricle has been identified as a mechanism contributing to diastolic filling [13] and recently shown to be associated with survival in patients with heart failure with preserved ejection fraction [14]. As long as the atrial short-axis area is smaller than the ventricular short-axis area, this geometrical relationship yields a net hydraulic force acting on the atrioventricular plane pushing it toward the atrium, thus aiding diastolic filling. The difference between the ventricular and atrial areas is denoted the atrioventricular area difference (AVAD) (Fig. 1) [15]. Since patients with rToF and pulmonary regurgitation (PR) have an underfilled left ventricle due to regurgitation and right ventricular dilatation [16], an altered geometrical relationship between the left atrium and ventricle may be expected. With a smaller left ventricle, the hydraulic force during diastole may be decreased or even reversed in direction, thereby impeding diastolic filling.

The aim was to assess AVAD and to determine whether the hydraulic force aids or impedes diastolic filling in patients with rToF and pulmonary regurgitation, compared to controls.

## Methods

### Study Design

This study was designed as a retrospective case–control study at a tertiary center for congenital heart defects. The Ethical Review board in Lund, Sweden, approved the study. The study followed the Declaration of Helsinki and written consent was obtained from all participants or their legal guardians before inclusion. Results are presented according to STROBE guidelines for observational studies [17].

Patients were considered eligible if having undergone cardiac magnetic resonance (CMR) imaging including cine images of the left atrium and ventricle after rToF.

Healthy controls underwent CMR as part of studies on healthy cardiac physiology and chosen for the current study by matching to patients’ age, with children being age matched with a range of − 1 to + 2 years, IQR 0.5 years, and adults with a range of 0–4 years, IQR 2 years. All had normal CMR findings and no reported cardiac conditions.

### Cardiac Magnetic Resonance Imaging

Cardiac magnetic resonance imaging with retrospective ECG gating was performed in the supine position using one of two 1.5 T scanners (Achieva, Philips Healthcare, Best, the Netherlands; or Aera, Siemens Healthineers, Erlangen, Germany). Short‐axis balanced steady‐state free‐precession images covering the whole heart were acquired. Images were analyzed in the software Segment (http://segment.heiberg.se) [18]. Endocardial borders of the left ventricle were semi-automatically delineated in all timeframes from end systole to end diastole, with manual corrections if needed. Left atrial borders including atrial appendages were manually delineated. Right ventricular endocardium was semi-automatically delineated, with manual corrections if needed, at end systole and end diastole. Two-dimensional free-breathing through-plane phase-contrast imaging was performed in the aorta and pulmonary artery in patients with rToF to quantify effective stroke volume and pulmonary regurgitation. Linear phase background correction was applied. All measurements were done in consensus with two observers (**and**, with 11 and 23 years of CMR experience, respectively).

Atrioventricular area difference was calculated as the largest ventricular short-axis area minus the largest atrial short-axis area at every timeframe from end systole to end diastole, in all subjects [13, 15]. The hydraulic force supports the motion of the atrioventricular plane when the mitral valve is open during diastole and the atrial and ventricular pressures are close to equal. Thus, for didactic purpose when discussing AVAD, end systole is denoted “beginning of diastole” throughout this work.

$$\text{AVAD}\left( {\text{cm}^{2} } \right)\, = \text{ventricular short-axis area}\,\left( {\text{cm}^{2} } \right){-}\,{\text{atrial short-axis area}}\,(\text{cm}^{2}).$$

Beginning of diastole and end diastole were set as the timeframes with the smallest and largest volume of the left ventricle and thus represent ventricular beginning of diastole and end diastole and not atrial time-points. Atrioventricular area difference was indexed to height (AVADi) to account for cardiovascular growth in the pediatric population while avoiding bias due to variation in body mass index [19].

### Statistical analysis

Statistical analyses were performed in GraphPad Prism (version 9.5.1, Boston, Massachusetts, USA). Gaussian distribution was not assumed and values are presented as median [interquartile range]. Groups were compared using Mann–Whitney’s unpaired test. Non-parametric Spearman’s test tested for correlation between variables. *p* values < 0.05 were considered to indicate statistically significant differences.

## Results

**Fig. 2:**
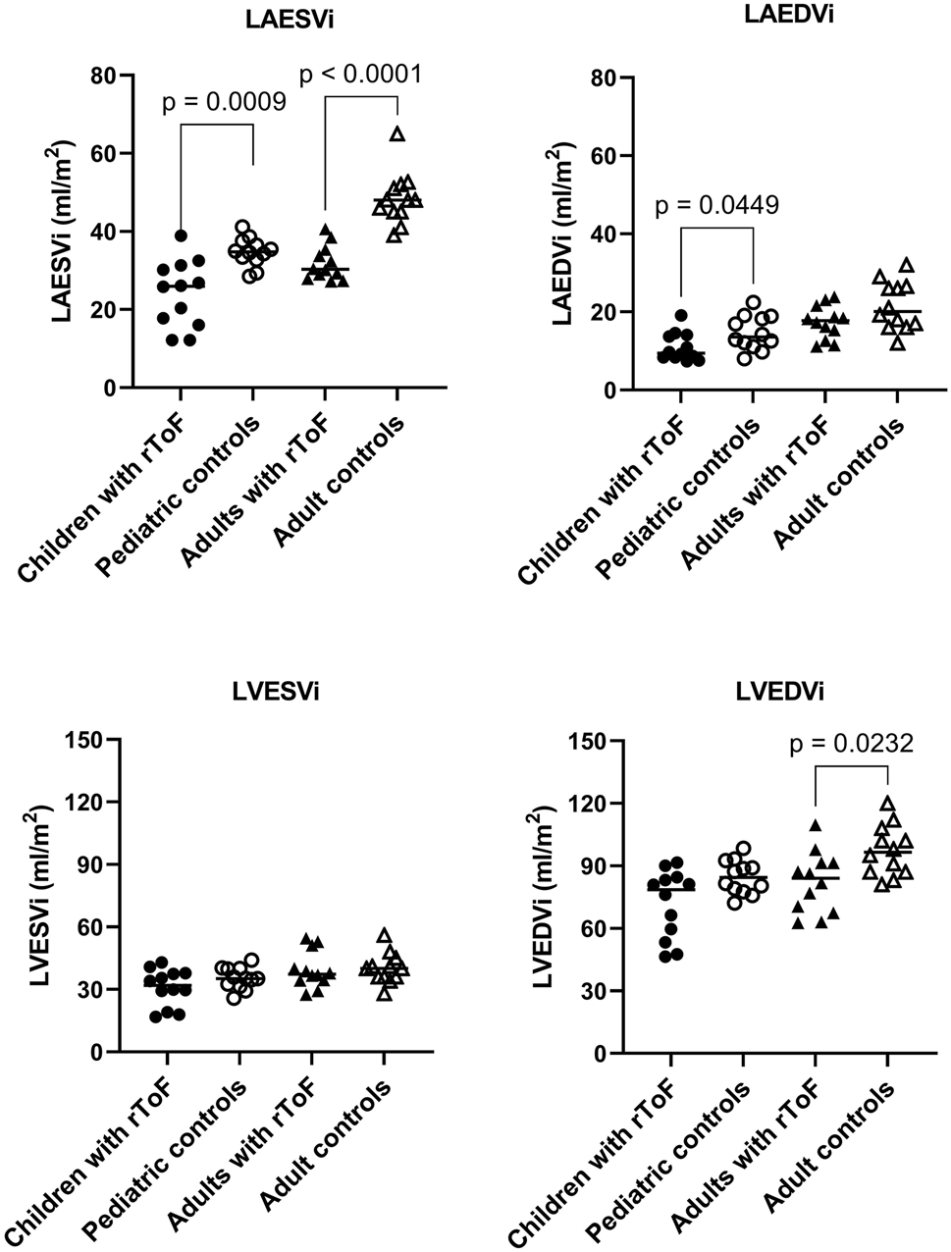
Left atrial and ventricular volumes at end systole and end diastole. Children with rToF had smaller left atrial volumes compared to controls. Adults with rToF had smaller left atrial volume at end systole compared to controls and also smaller left ventricular end-diastolic volumes compared to controls. *LVESVi* Left ventricular end-systolic volume indexed to body surface area, *LVEDVi* Left ventricular end-diastolic volume indexed to body surface area, *rToF* repaired Tetralogy of Fallot

Table 1 shows subject characteristics. Table 2 and Fig. 2 show cardiac volumes and function for children and adults with rToF compared to controls. Table 3 show AVADi for all groups. All participants with rToF had pulmonary regurgitation. One adult with rToF had mild mitral regurgitation. This same participant also had the largest left atrial end-systolic volume indexed to body surface area (BSA), the second largest left ventricular end-systolic and largest end-diastolic volumes indexed to BSA, and the largest AVADi at beginning of diastole. One child with rToF had current treatment with beta-blockers, all others were without medication. Nine children with rToF (75%) were classified as New York Heart Association (NYHA) class I and 3 children with rToF (25%) as NYHA class II. Ten adult patients with rToF (83%) were classified as NYHA class I and the remaining 2 adult patients (17%) as NYHA class II. One adult patient with rToF was treated with acetylsalicylic acid, all others were without medication.

**Table 1:** Patient characteristics

	Children with rToF	Pediatric controls	Adults with rToF	Adult controls
Number of subjects (female)	12 (5)	12 (4)	12 (4)	12 (5)
Age at CMR, years	11.5 [9–13]	10.5 [8.5–13]	21.5 [19–27]	24 [22–29]
Height, cm	148 [137–158]	146 [136–162]	173 [169–176]	176 [171–181]
Weight, kg	43 [37–47]	39 [31–47]	70 [64–77]	70 [64–74]
BSA, m^2^	1.3 [1.2–1.4]	1.2 [1.1–1.5]	1.8 [1.7–1.9]	1.9 [1.8–1.9]
BMI, kg/m^2^	19.0 [17.5–21.4]	16.6 [15.4–18.4]	23.4 [21.9–26.1]	22.3 [20.9–24.2]
QRS duration, ms	131 [119–140]	NA	160 [147–173]	NA
Age at primary correction, months	3 [2–8]	NA	11 [8.5–17.5] (*n* = 11)	NA
Time from primary correction to CMR, years	10 [9–12]	NA	18 [15–122] (*n* = 11)	NA

*rToF*  repaired Tetralogy of Fallot, *CMR* Cardiac Magnetic Resonance, *BSA* Body Surface Area, Data is presented as median [interquartile range]

**Table 2:** Cardiac volumes and function

	Children with rToF (*n* = 12)	Pediatric controls (*n* = 12)		Adults with rToF (*n* = 12)	Adult controls (*n* = 12)	
HR, beats/min	77 [74–80]	70 [67–78]	*p* = 0.22	71 [66–74]	63 [49–74]	*p* = 0.39
SBP, mmHg	110 [105–120]	100 [95–105] (n = 11)	*p* = 0.0032	120 [110–125]	120 [120–130]	*p* = 0.34
DBP, mmHg	65 [60–70]	60 [55–60] (n = 11)	*p* = 0.076	70 [65–80]	70 [70–75]	*p* = 0.91
Left ventricle						
EDVi, ml/m^2^	79 [58–84]	85 [79–90]	*p* = 0.089	84 [70–91]	97 [87–104]	*p* = 0.023
EDV indexed to height, ml/m	69 [42–78]	72 [62–78]	*p* = 0.38	86 [78–94]	99 [95–113]	*p* = 0.017
ESVi, ml/m^2^	32 [27–37]	35 [32–40]	*p* = 0.35	37 [34–43]	40 [36–42]	*p *= 0.70
ESV indexed to height, ml/m	28 [23–55]	28 [26–35]	*p* = 0.98	39 [37–49]	43 [37–45]	*p* = 0.93
SVi, ml/m^2^	40 [36–48]	50 [44–53]	*p* = 0.045	40 [36–51]	54 [51–63]	*p* = 0.0027
SV indexed to height, ml/m	36 [28–44]	41 [36–49]	*p* = 0.16	41 [39–53]	58 [56–66]	*p* = 0.0009
EF, %	58 [54–60]	58 [55–62]	*p* = 0.80	52 [49–57]	59 [55–63]	*p* = 0.0068
CI, L/min/ m^2^	3.1 [2.6–3.7]	3.4 [3.3–3.9]	*p* = 0.16	2.9 [2.6–3.4]	3.6 [2.9–3.9]	*p* = 0.10
Left atrium						
Volume at ventricular end-diastole indexed to BSA, ml/m^2^	9 [8–14]	14 [12–18]	*p* = 0.045	18 [15–19]	20 [17–26]	*p *= 0.12
Volume at ventricular end-diastole indexed to height, ml/m	8 [7–12]	12 [10–15]	*p* = 0.068	19 [15–22]	22 [18–28]	*p* = 0.10
Volume at ventricular end-systole indexed to BSA, ml/m^2^	26 [17–30]	35 [33–37]	*p* = 0.0009	30 [29–34]	48 [45–51]	*p* < 0.0001
Volume at ventricular end-systole indexed to height, ml/m	21 [15–28]	29 [25–33]	*p* = 0.021	33 [30–40]	51 [49–53]	*p* < 0.0001
Right ventricle						
EDVi, ml/m^2^	130 [110–135]	98 [88–103]	*p* = 0.0001	148 [120–163]	115 [108–122]	*p* = 0.045
EDV indexed to height, ml/m	112 [94–122]	87 [71–91]	*p* = 0.0005	154 [133–174]	121 [113–131]	*p *= 0.012
ESVi, ml/m^2^	64 [60–70]	43 [40–48]	*p* < 0.0001	94 [77–101]	57 [54–59]	*p* < 0.0001
ESV indexed to height, ml/m	58 [49–64]	39 [33–43]	*p* < 0.0001	93 [80–117]	57 [55–65]	*p* < 0.0001
EF, %	46 [44–52]	53 [50–55]	*p* = 0.052	37 [33–42]	51 [48–52]	*p* < 0.0001
PR fraction, %	39 [36–47]	NA		38 [26–44]	NA	

*rToF* repaired Tetralogy of Fallot, *HR* Heart rate, *SBP* = Systolic Blood Pressure, *DB*P Diastolic Blood Pressure, *EDVi* End-diastolic volume indexed to body surface area, *ESVi* End-systolic volume indexed to body surface area, *SVi* Stroke volume indexed to body surface area, *EF* Ejection fraction, *CI* Cardiac index, *PR* pulmonary regurgitation, Data are presented as median [interquartile range]

**Table 3:** Atrioventricular area difference at beginning of diastole and end diastole

	Children with rToF (*n* = 12)	Pediatric controls (*n* = 12)		Adults with rToF (*n* = 12)	Adult controls (*n* = 12)	
AVADi Beginning of diastole, cm^2^/m	0.3 [− 1.3 to 0.8]	− 2.7 [− 4.9 to − 1.7]	*p* = 0.015	− 0.6 [− 1.5 to − 0.2]	− 3.3 [− 3.8 to − 2.8]	*p* = 0.036
AVADi End diastole, cm^2^/m	10.3 [7.4 to 12.7]	10.5 [9.3 to 11.7]	*p* = 0.75	8.4 [7.6 to 9.4]	9.5 [8.3 to 11.4]	*p* = 0.11
Time of diastole with AVADi > 0, %	94 [78–100]	76 [74–82]	*p *= 0.14	85 [75–95]	80 [78–87]	*p* = 0.50

Atrioventricular area difference (AVAD) indexed to height, presented at beginning of diastole and end diastole. *rToF* repaired Tetralogy of Fallot. *AVADi* Atrioventricular Area Difference indexed to height. Data is presented as median [interquartile range]

Body mass index variation in the pediatric population was 14.9–23.7 kg/m^2^ for children with rToF and 14.0–24.3 kg/m^2^ for controls. Ranges for body mass index z-scores in the pediatric population were − 2.1 to 2.1 for children with rToF and − 2.0 to 1.5 for controls. Two pediatric controls and four children with rToF were classified as overweight (BMI z-scores 1.4, 1.5, 1.1, 1.1, 1.6, 1.7). One child with rToF was classified as obese (BMI z-score 2.1).

Atrioventricular area difference.

**Fig. 3:**
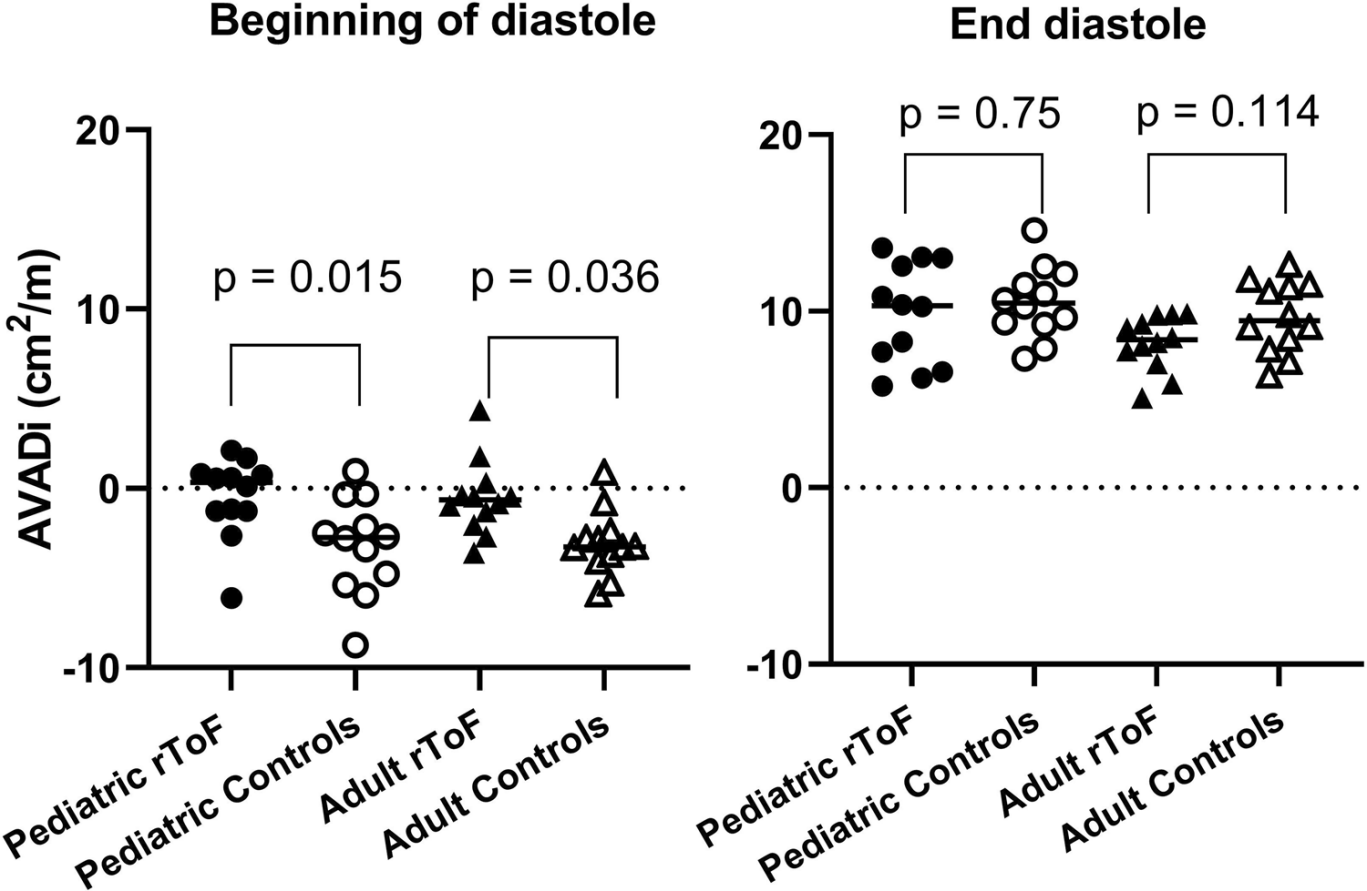
Atrioventricular area difference indexed to height at beginning of diastole and at end diastole. Children and adults with rToF have a sustained atrioventricular area difference and thus a net hydraulic force contributing to diastolic filling, similar to that of healthy controls, despite a small underfilled left ventricle due to pulmonary regurgitation. *AVADi* Atrioventricular area difference indexed, *rToF*  repaired Tetralogy of Fallot

Table 3 shows AVADi at beginning of ventricular diastole and at end systole as well as percentage of diastole spent with AVADi > 0 cm^2^/m. Figure 3 shows that AVADi was higher at beginning of diastole in children with rToF (0.3 cm^2^/m [− 1.3 to 0.8]) than in controls (− 2.7 cm^2^/m [− 4.9 to − 1.7], p = 0.015). Also, adults with rToF had a higher AVADi at beginning of diastole (− 0.6 [− 1.5 to − 0.2]) compared to controls (− 3.3 cm^2^/m [− 3.8 to − 2.8], *p* = 0.036). No difference was found in AVADi at end diastole for either children or adults with rToF compared to respective controls. No difference was found when comparing percentage of diastole spent with AVADi > 0 between groups.

There was no correlation between degree of pulmonary regurgitation in patients with rToF and AVADi, either at beginning of diastole (children: *p* = 0.42, adults: *p* = 0.69) or at end diastole (children: *p* = 0.97, adults: *p* = 0.54).

There was no correlation between right ventricular end-diastolic volume indexed to BSA and AVADi at end diastole in patients with rToF (children: *p* = 0.12, adults: *p* = 0.33). Furthermore, there was no correlation between left ventricular end-diastolic volume indexed to BSA and AVADi at end diastole in patients with rToF (children: *p* = 0.77, adults: *p* = 0.14).

## Discussion

The main finding of this study is that the geometrical relationship between the left atrium and left ventricle is normal in children and adults with rToF and pulmonary regurgitation up to 18 [15–19] years after surgical repair, compared to healthy controls. Thus, the direction of the hydraulic force, which is dependent on this geometrical relationship and assessed by AVAD, is sustained and contributes to left ventricular diastolic filling. This is despite the underfilled left ventricle seen in these patients with rToF secondary to pulmonary regurgitation and right ventricular dilatation.

The current study showed that both children and adults with rToF had higher AVADi at the start of diastole compared to controls. However, when comparing duration of diastole with AVADi > 0, i.e., a hydraulic force contributing to diastolic filling, there was no difference between patients and controls. This supports that patients with rToF have a sustained AVAD and a hydraulic force contributing to diastolic filling of the left ventricle. The greater AVADi at start of diastole (i.e., end systole) in children and adults with rToF could be an effect of pulmonary regurgitation that during systole would lead to a smaller ejected volume into the pulmonary artery. This would in turn lead to a concomitant decrease in left atrial area at end systole and thus increased AVADi. At end diastole the geometrical relationship is however preserved.

In comparison, in patients with heart failure with preserved ejection fraction, who also have small left ventricles, AVAD and thus the hydraulic force is low and does not aid diastolic filling [15]. Thus, patients with heart failure with preserved ejection fraction and patients with rToF with pulmonary regurgitation are similar in that the left ventricle is small in both, however the groups differ in AVAD. These somewhat contradicting results can be explained by the pathophysiological differences between patients with heart failure with preserved ejection fraction and rToF with pulmonary regurgitation where the latter exhibit an underfilling of the left ventricle due to regurgitation and has a concomitantly small left atrium and therefore no effect on AVAD is found. On the contrary, patients with heart failure with preserved ejection fraction have smaller ventricles due to lower compliance and high resistance to filling, resulting in a large atrium and thereby lower AVAD. The effects on AVAD and net hydraulic force of having a disproportionately large atrium have also been shown in heart-transplanted patients [20].

The finding of small left ventricular volumes both in children and adults with rToF in the current study is in line with previous studies on left ventricular function in rToF [9, 16]. Also, left atrial volumes were smaller in patients with rToF in the current study, at beginning of diastole for both children and adults and at end diastole for children, compared to healthy controls. Previous reports have been inconsistent regarding atrial volumes in patients with rToF [12, 21–24]. The small atrial volumes found in the current study can be explained firstly by the young age at which patients with rToF underwent primary repair, median 3 months for children and 9 months for adults. Indeed, two previous studies have concluded that older age at primary repair is associated with greater risk of atrial dilatation [12, 21]. Secondly, subjects in the current study were young at time of CMR, median age 11.5 years for children and 21 years for adults. In the studies reporting dilated left atria, median age at CMR were 28, 35 and 37 years [12, 21, 24].

In the current study AVADi was not related to either left or right ventricular end-diastolic volume, nor was there a relationship between AVADi and degree of pulmonary regurgitation. This highlights the fact that AVAD and the direction of the hydraulic force supporting diastolic filling is dependent on short-axis area differences and not necessarily affected by the simultaneous decrease in left atrial and ventricular volumes which in rToF is due to pulmonary regurgitation.

In this study, one adult with rToF had mild mitral regurgitation whilst maintaining a positive AVADi and a hydraulic force aiding diastolic filling. Mitral regurgitation may lead to larger left atrial and ventricular volumes. However, as long as the relationship between atrial and ventricular area does not change, this will not alter the AVAD and thus the direction of the hydraulic force will be sustained.

In patients with heart failure with preserved ejection fraction, AVAD was a prognostic marker for survival and was proposed to carry information regarding diastolic function beyond traditional measurements [14]. Measures of left sided cardiac function is relevant in rToF as there is evidence that left ventricular function is a strong predictor of ventricular arrhythmias, major adverse events and impaired exercise tolerance [5, 10, 25–27]. Additionally, left atrial function is associated with risk of major cardiovascular events in these patients [12, 21]. Atrioventricular area difference is a mechanism that, when altered, would lead to a disturbed diastolic function which may explain the proposed prognostic value of AVAD [14]. The current study did not address the prognostic value of AVAD in patients with rToF. Although these patients exhibited normal AVAD compared to controls repeated measurements over time may uncover diastolic dysfunction at an early stage. When and how often these measurements are to be made needs to be addressed in a longitudinal follow-up study.

## Limitations

The study population may be considered small. However, CMR has high precision in measuring differences in cardiac volumes why the numbers are sufficient to show differences in AVADi [28]. As invasive pressure measurements were not performed the exact magnitude of the hydraulic force was not determined. Echocardiographic data was not obtained in this study.

## Conclusion

Children and adults with rToF and pulmonary regurgitation have an atrioventricular area difference that do not differ from controls and thus a net hydraulic force that contributes to left ventricular diastolic filling, despite a small underfilled left ventricle due to pulmonary regurgitation.

## References

[1] Ylitalo P, Nieminen H, Pitkänen OM, Jokinen E, Sairanen H (2015) Need of transannular patch in tetralogy of fallot surgery carries a higher risk of reoperation but has no impact on late survival: results of Fallot repair in Finland. Eur J Cardiothorac Surg 48:91–97 25326015 10.1093/ejcts/ezu401

[2] Sarris GE, Comas JV, Tobota Z, Maruszewski B (2012) Results of reparative surgery for tetralogy of Fallot: data from the european association for cardio-thoracic surgery congenital database. Eur J Cardiothorac Surg 42:766–774 23087090 10.1093/ejcts/ezs478

[3] Jacobs JP, Mayer JE, Pasquali SK, Hill KD, Overman DM, Louis JD et al (2018) The society of thoracic surgeons congenital heart surgery database: 2018 update on outcomes and quality. Ann Thorac Surg. 10.1016/j.athoracsur.2018.01.001 29337121 10.1016/j.athoracsur.2018.01.001

[4] Mueller AS, McDonald DM, Singh HS, Ginns JN (2020) Heart failure in adult congenital heart disease: tetralogy of Fallot. Heart Fail Rev 25:583–598 31925611 10.1007/s10741-019-09903-0

[5] Valente AM, Gauvreau K, Assenza GE, Babu-Narayan SV, Schreier J, Gatzoulis MA et al (2014) Contemporary predictors of death and sustained ventricular tachycardia in patients with repaired tetralogy of Fallot enrolled in the INDICATOR cohort. Heart 100:247–253 24179163 10.1136/heartjnl-2013-304958 PMC3913216

[6] Ghonim S, Gatzoulis MA, Ernst S, Li W, Moon JC, Smith GC et al (2022) predicting survival in repaired tetralogy of Fallot. JACC Cardiovasc Imaging 15:257–268 34656466 10.1016/j.jcmg.2021.07.026 PMC8821017

[7] Baumgartner H, De Backer J, Babu-Narayan SV, Budts W, Chessa M, Diller G-P et al (2021) 2020 ESC Guidelines for the management of adult congenital heart disease. Eur Heart J 42:563–645 32860028 10.1093/eurheartj/ehaa554

[8] Gatzoulis MA, Balaji S, Webber SA, Siu SC, Hokanson JS, Poile C et al (2000) Risk factors for arrhythmia and sudden cardiac death late after repair of tetralogy of Fallot: a multicentre study. The Lancet 356:975–981 10.1016/S0140-6736(00)02714-8 11041398

[9] Friedberg MK, Fernandes FP, Roche SL, Grosse-Wortmann L, Manlhiot C, Fackoury C et al (2012) Impaired right and left ventricular diastolic myocardial mechanics and filling in asymptomatic children and adolescents after repair of tetralogy of Fallot. Eur Heart J—Cardiovasc Imaging 13:905–913 22467442 10.1093/ehjci/jes067

[10] Aboulhosn JA, Lluri G, Gurvitz MZ, Khairy P, Mongeon F-P, Kay J et al (2013) Left and right ventricular diastolic function in adults with surgically repaired tetralogy of Fallot: a multi-institutional study. Can J Cardiol 29:866–872 23369488 10.1016/j.cjca.2012.11.003

[11] Andrade AC, Jerosch-Herold M, Wegner P, Gabbert DD, Voges I, Pham M et al (2019) Determinants of left ventricular dysfunction and remodeling in patients with corrected tetralogy of Fallot. J Am Heart Assoc 8:e009618 31474177 10.1161/JAHA.118.009618 PMC6755839

[12] Ait Ali L, Lurz P, Ripoli A, Rossi G, Kister T, Aquaro GD et al (2019) Implications of atrial volumes in surgical corrected tetralogy of Fallot on clinical adverse events. Int J Cardiol 283:107–111 30819586 10.1016/j.ijcard.2019.02.018

[13] Maksuti E, Carlsson M, Arheden H, Kovács SJ, Broomé M, Ugander M (2017) Hydraulic forces contribute to left ventricular diastolic filling. Sci Rep 7:43505 28256604 10.1038/srep43505 PMC5334655

[14] Soundappan D, Fung ASY, Loewenstein DE, Playford D, Strange G, Kozor R et al (2023) Decreased diastolic hydraulic forces incrementally associate with survival beyond conventional measures of diastolic dysfunction. Sci Rep 13:16396 37773251 10.1038/s41598-023-41694-1 PMC10541860

[15] Steding-Ehrenborg K, Hedström E, Carlsson M, Maksuti E, Broomé M, Ugander M et al (2021) Hydraulic force is a novel mechanism of diastolic function that may contribute to decreased diastolic filling in HFpEF and facilitate filling in HFrEF. J Appl Physiol 130:993–1000 33539261 10.1152/japplphysiol.00890.2020

[16] Ylitalo P, Jokinen E, Lauerma K, Holmström M, Pitkänen-Argillander OM (2018) Additional mechanism for left ventricular dysfunction: chronic pulmonary regurgitation decreases left ventricular preload in patients with tetralogy of Fallot. Cardiol Young 28:208–213 29019299 10.1017/S1047951117001457

[17] Von Elm E, Altman DG, Egger M, Pocock SJ, Gøtzsche PC, Vandenbroucke JP (2008) The strengthening the reporting of observational studies in epidemiology (STROBE) statement: guidelines for reporting observational studies. J Clin Epidemiol 61:344–349 18313558 10.1016/j.jclinepi.2007.11.008

[18] Heiberg E, Sjögren J, Ugander M, Carlsson M, Engblom H, Arheden H (2010) Design and validation of segment—freely available software for cardiovascular image analysis. BMC Med Imaging 10:1 20064248 10.1186/1471-2342-10-1 PMC2822815

[19] Mahgerefteh J, Lai W, Colan S, Trachtenberg F, Gongwer R, Stylianou M et al (2021) Height versus body surface area to normalize cardiovascular measurements in children using the pediatric heart network echocardiographic Z-score database. Pediatr Cardiol 42:1284–1292 33877418 10.1007/s00246-021-02609-x PMC8684290

[20] Steding-Ehrenborg K, Nelsson A, Hedström E, Engblom H, Ingvarsson A, Nilsson J, Braun O, Arheden H. Diastolic filling in patients after heart transplantation is impaired due to an altered geometrical relationship between the left atrium and ventricle. Accepted for JAHA 12 march 2024 10.1161/JAHA.123.033672 PMC11255639 38780152

[21] Baggen VJM, Schut A-RW, Cuypers JAAE, Witsenburg M, Boersma E, Van Den Bosch AE et al (2017) Prognostic value of left atrial size and function in adults with tetralogy of Fallot. Int J Cardiol 236:125–131 28268081 10.1016/j.ijcard.2017.02.153

[22] Hu L, Ouyang R, Liu X, Shuang L, Xiaodan Z, Guo C et al (2021) Impairment of left atrial function in pediatric patients with repaired tetralogy of Fallot: a cardiovascular magnetic resonance imaging study. Int J Cardiovasc Imaging 37:3255–3267 34105081 10.1007/s10554-021-02302-3

[23] Riesenkampff E, Mengelkamp L, Mueller M, Kropf S, Abdul-Khaliq H, Sarikouch S et al (2010) Integrated analysis of atrioventricular interactions in tetralogy of Fallot. Am J Physiol-Heart Circ Physiol 299:H364–H371 20495149 10.1152/ajpheart.00264.2010 PMC2930385

[24] Egbe AC, Banala K, Vojjini R, Jadav R, Sufian M, Pellikka PA et al (2020) Left ventricular filling pressure in tetralogy of Fallot: correlation between invasive and noninvasive indices. IJC Heart Vasc 26:100457 10.1016/j.ijcha.2019.100457 PMC6938956 31909179

[25] Knauth AL, Gauvreau K, Powell AJ, Landzberg MJ, Walsh EP, Lock JE et al (2008) Ventricular size and function assessed by cardiac MRI predict major adverse clinical outcomes late after tetralogy of Fallot repair. Heart 94:211–216 17135219 10.1136/hrt.2006.104745

[26] Diller G-P, Kempny A, Liodakis E, Alonso-Gonzalez R, Inuzuka R, Uebing A et al (2012) Left ventricular longitudinal function predicts life-threatening ventricular arrhythmia and death in adults with repaired tetralogy of Fallot. Circulation 125:2440–2446 22496160 10.1161/CIRCULATIONAHA.111.086983

[27] Steinmetz M, Stümpfig T, Seehase M, Schuster A, Kowallick J, Müller M et al (2021) Impaired exercise tolerance in repaired tetralogy of fallot is associated with impaired biventricular contractile reserve: an exercise-stress real-time cardiovascular magnetic resonance study. Circ Cardiovasc Imaging 14:e011823 34384226 10.1161/CIRCIMAGING.120.011823

[28] Bellenger N, Davies LC, Francis J, Coats A, Pennell D (2000) Reduction in sample size for studies of remodeling in heart failure by the use of cardiovascular magnetic resonance. J Cardiovasc Magn Reson 2:271–278 11545126 10.3109/10976640009148691

