# Correction: Atrioventricular Area Difference Aids Diastolic Filling in Patients with Repaired Tetralogy of Fallot

**DOI:** 10.1007/s00246-025-03811-x

**Published:** 2025-03-17

**Authors:** Martin Johansson, Erik Hedström, Katarina Steding-Ehrenborg, Misha Bhat, Petru Liuba, Håkan Arheden, Pia Sjöberg

**Affiliations:** 1 Clinical Physiology, Department of Clinical Sciences Lund, Lund University, Lund, Sweden; 2 Department of Clinical Physiology, Skåne University Hospital, 22185 Lund, Sweden; 3 Department of Pediatric Anesthesia and Intensive Care, Skåne University Hospital, Lund, Sweden; 4 Diagnostic Radiology, Department of Clinical Sciences Lund, Lund University, Lund, Sweden; 5 Department of Radiology, Skåne University Hospital, Lund, Sweden; 6 Pediatrics, Department of Clinical Sciences Lund, Lund University, Lund, Sweden; 7 Pediatric Cardiology, Children’s Heart Centre, Skåne University Hospital, Lund, Sweden

**Correction to: Pediatric Cardiology** 10.1007/s00246-024-03508-7

In this article, the affiliation ‘Diagnostic Radiology, Department of Clinical Sciences Lund, Lund University, Lund, Sweden’ was incorrectly tagged for the author ‘Martin Johansson’ and should have been removed.

The sixth affiliation was incorrectly published as ‘Department of Clinical Sciences Lund, Lund University, Lund, Sweden’ but should have been ‘Pediatrics, Department of Clinical Sciences Lund, Lund University, Lund, Sweden’.

In Table 2, the data in the row ‘Volume at ventricular end-systole indexed to height, ml/m’ and column ‘Children with rToF (*n* = 12)’ was incorrectly written as ‘21 eee’ but should have been ‘21 [15–28]’. The incorrect and the corrected versions of Table 2 are given below.

**Incorrect Table:**

**Table 2:** Cardiac volumes and function

	Children with rToF (*n* = 12)	Pediatric controls (*n* = 12)		Adults with rToF (*n* = 12)	Adult controls (*n* = 12)	
HR, beats/min	77 [74–80]	70 [67–78]	*p* = 0.22	71 [66–74]	63 [49–74]	*p* = 0.39
SBP, mmHg	110 [105–120]	100 [95–105] (n = 11)	*p* = 0.0032	120 [110–125]	120 [120–130]	*p* = 0.34
DBP, mmHg	65 [60–70]	60 [55–60] (n = 11)	*p* = 0.076	70 [65–80]	70 [70–75]	*p* = 0.91
Left ventricle						
EDVi, ml/m^2^	79 [58–84]	85 [79–90]	*p* = 0.089	84 [70–91]	97 [87–104]	*p* = 0.023
EDV indexed to height, ml/m	69 [42–78]	72 [62–78]	*p* = 0.38	86 [78–94]	99 [95–113]	*p* = 0.017
ESVi, ml/m^2^	32 [27–37]	35 [32–40]	*p* = 0.35	37 [34–43]	40 [36–42]	*p *= 0.70
ESV indexed to height, ml/m	28 [23–55]	28 [26–35]	*p* = 0.98	39 [37–49]	43 [37–45]	*p* = 0.93
SVi, ml/m^2^	40 [36–48]	50 [44–53]	*p* = 0.045	40 [36–51]	54 [51–63]	*p* = 0.0027
SV indexed to height, ml/m	36 [28–44]	41 [36–49]	*p* = 0.16	41 [39–53]	58 [56–66]	*p* = 0.0009
EF, %	58 [54–60]	58 [55–62]	*p* = 0.80	52 [49–57]	59 [55–63]	*p* = 0.0068
CI, L/min/ m^2^	3.1 [2.6–3.7]	3.4 [3.3–3.9]	*p* = 0.16	2.9 [2.6–3.4]	3.6 [2.9–3.9]	*p* = 0.10
Left atrium						
Volume at ventricular end-diastole indexed to BSA, ml/m^2^	9 [8–14]	14 [12–18]	*p* = 0.045	18 [15–19]	20 [17–26]	*p *= 0.12
Volume at ventricular end-diastole indexed to height, ml/m	8 [7–12]	12 [10–15]	*p* = 0.068	19 [15–22]	22 [18–28]	*p* = 0.10
Volume at ventricular end-systole indexed to BSA, ml/m^2^	26 [17–30]	35 [33–37]	*p* = 0.0009	30 [29–34]	48 [45–51]	*p* < 0.0001
Volume at ventricular end-systole indexed to height, ml/m	21 eeee	29 [25–33]	*p* = 0.021	33 [30–40]	51 [49–53]	*p* < 0.0001
Right ventricle						
EDVi, ml/m^2^	130 [110–135]	98 [88–103]	*p* = 0.0001	148 [120–163]	115 [108–122]	*p* = 0.045
EDV indexed to height, ml/m	112 [94–122]	87 [71–91]	*p* = 0.0005	154 [133–174]	121 [113–131]	*p *= 0.012
ESVi, ml/m^2^	64 [60–70]	43 [40–48]	*p* < 0.0001	94 [77–101]	57 [54–59]	*p* < 0.0001
ESV indexed to height, ml/m	58 [49–64]	39 [33–43]	*p* < 0.0001	93 [80–117]	57 [55–65]	*p* < 0.0001
EF, %	46 [44–52]	53 [50–55]	*p* = 0.052	37 [33–42]	51 [48–52]	*p* < 0.0001
PR fraction, %	39 [36–47]	NA		38 [26–44]	NA	

*rToF* repaired Tetralogy of Fallot, *HR* Heart rate, *SBP* = Systolic Blood Pressure, *DB*P Diastolic Blood Pressure, *EDVi* End-diastolic volume indexed to body surface area, *ESVi* End-systolic volume indexed to body surface area, *SVi* Stroke volume indexed to body surface area, *EF* Ejection fraction, *CI* Cardiac index, *PR* pulmonary regurgitation, Data are presented as median [interquartile range]

**Correct Table:**

**Table 2:** Cardiac volumes and function

	Children with rToF (*n* = 12)	Pediatric controls (*n* = 12)		Adults with rToF (*n* = 12)	Adult controls (*n* = 12)	
HR, beats/min	77 [74–80]	70 [67–78]	*p* = 0.22	71 [66–74]	63 [49–74]	*p* = 0.39
SBP, mmHg	110 [105–120]	100 [95–105] (n = 11)	*p* = 0.0032	120 [110–125]	120 [120–130]	*p* = 0.34
DBP, mmHg	65 [60–70]	60 [55–60] (n = 11)	*p* = 0.076	70 [65–80]	70 [70–75]	*p* = 0.91
Left ventricle						
EDVi, ml/m^2^	79 [58–84]	85 [79–90]	*p* = 0.089	84 [70–91]	97 [87–104]	*p* = 0.023
EDV indexed to height, ml/m	69 [42–78]	72 [62–78]	*p* = 0.38	86 [78–94]	99 [95–113]	*p* = 0.017
ESVi, ml/m^2^	32 [27–37]	35 [32–40]	*p* = 0.35	37 [34–43]	40 [36–42]	*p *= 0.70
ESV indexed to height, ml/m	28 [23–55]	28 [26–35]	*p* = 0.98	39 [37–49]	43 [37–45]	*p* = 0.93
SVi, ml/m^2^	40 [36–48]	50 [44–53]	*p* = 0.045	40 [36–51]	54 [51–63]	*p* = 0.0027
SV indexed to height, ml/m	36 [28–44]	41 [36–49]	*p* = 0.16	41 [39–53]	58 [56–66]	*p* = 0.0009
EF, %	58 [54–60]	58 [55–62]	*p* = 0.80	52 [49–57]	59 [55–63]	*p* = 0.0068
CI, L/min/ m^2^	3.1 [2.6–3.7]	3.4 [3.3–3.9]	*p* = 0.16	2.9 [2.6–3.4]	3.6 [2.9–3.9]	*p* = 0.10
Left atrium						
Volume at ventricular end-diastole indexed to BSA, ml/m^2^	9 [8–14]	14 [12–18]	*p* = 0.045	18 [15–19]	20 [17–26]	*p *= 0.12
Volume at ventricular end-diastole indexed to height, ml/m	8 [7–12]	12 [10–15]	*p* = 0.068	19 [15–22]	22 [18–28]	*p* = 0.10
Volume at ventricular end-systole indexed to BSA, ml/m^2^	26 [17–30]	35 [33–37]	*p* = 0.0009	30 [29–34]	48 [45–51]	*p* < 0.0001
Volume at ventricular end-systole indexed to height, ml/m	21 [15–28]	29 [25–33]	*p* = 0.021	33 [30–40]	51 [49–53]	*p* < 0.0001
Right ventricle						
EDVi, ml/m^2^	130 [110–135]	98 [88–103]	*p* = 0.0001	148 [120–163]	115 [108–122]	*p* = 0.045
EDV indexed to height, ml/m	112 [94–122]	87 [71–91]	*p* = 0.0005	154 [133–174]	121 [113–131]	*p *= 0.012
ESVi, ml/m^2^	64 [60–70]	43 [40–48]	*p* < 0.0001	94 [77–101]	57 [54–59]	*p* < 0.0001
ESV indexed to height, ml/m	58 [49–64]	39 [33–43]	*p* < 0.0001	93 [80–117]	57 [55–65]	*p* < 0.0001
EF, %	46 [44–52]	53 [50–55]	*p* = 0.052	37 [33–42]	51 [48–52]	*p* < 0.0001
PR fraction, %	39 [36–47]	NA		38 [26–44]	NA	

*rToF* repaired Tetralogy of Fallot, *HR* Heart rate, *SBP* = Systolic Blood Pressure, *DB*P Diastolic Blood Pressure, *EDVi* End-diastolic volume indexed to body surface area, *ESVi* End-systolic volume indexed to body surface area, *SVi* Stroke volume indexed to body surface area, *EF* Ejection fraction, *CI* Cardiac index, *PR* pulmonary regurgitation, Data are presented as median [interquartile range]

The original article has been corrected.

